# Time series analysis of human brucellosis in mainland China by using Elman and Jordan recurrent neural networks

**DOI:** 10.1186/s12879-019-4028-x

**Published:** 2019-05-14

**Authors:** Wei Wu, Shu-Yi An, Peng Guan, De-Sheng Huang, Bao-Sen Zhou

**Affiliations:** 1 Department of Epidemiology, School of Public Health, China Medical University, Shenyang, Liaoning China; 2 Liaoning Provincial Center for Disease Control and Prevention, Shenyang, Liaoning China; 3 Department of Mathematics, School of Fundamental Sciences, China Medical University, Shenyang, Liaoning China

**Keywords:** Time series analysis, Human brucellosis, Recurrent neural network

## Abstract

**Background:**

Establishing epidemiological models and conducting predictions seems to be useful for the prevention and control of human brucellosis. Autoregressive integrated moving average (ARIMA) models can capture the long-term trends and the periodic variations in time series. However, these models cannot handle the nonlinear trends correctly. Recurrent neural networks can address problems that involve nonlinear time series data. In this study, we intended to build prediction models for human brucellosis in mainland China with Elman and Jordan neural networks. The fitting and forecasting accuracy of the neural networks were compared with a traditional seasonal ARIMA model.

**Methods:**

The reported human brucellosis cases were obtained from the website of the National Health and Family Planning Commission of China. The human brucellosis cases from January 2004 to December 2017 were assembled as monthly counts. The training set observed from January 2004 to December 2016 was used to build the seasonal ARIMA model, Elman and Jordan neural networks. The test set from January 2017 to December 2017 was used to test the forecast results. The root mean squared error (RMSE), mean absolute error (MAE) and mean absolute percentage error (MAPE) were used to assess the fitting and forecasting accuracy of the three models.

**Results:**

There were 52,868 cases of human brucellosis in Mainland China from January 2004 to December 2017. We observed a long-term upward trend and seasonal variance in the original time series. In the training set, the RMSE and MAE of Elman and Jordan neural networks were lower than those in the ARIMA model, whereas the MAPE of Elman and Jordan neural networks was slightly higher than that in the ARIMA model. In the test set, the RMSE, MAE and MAPE of Elman and Jordan neural networks were far lower than those in the ARIMA model.

**Conclusions:**

The Elman and Jordan recurrent neural networks achieved much higher forecasting accuracy. These models are more suitable for forecasting nonlinear time series data, such as human brucellosis than the traditional ARIMA model.

**Electronic supplementary material:**

The online version of this article (10.1186/s12879-019-4028-x) contains supplementary material, which is available to authorized users.

## Background

Brucellosis is an anthropozoonosis caused by *Brucella melitensis* bacteria [1]. The occurrence of human brucellosis results from eating undercooked meat or drinking the unpasteurized milk of infected animals or coming in contact with their secretions. The epidemiological characteristics of brucellosis in industrialized countries have undergone dramatic changes over the past few decades. Brucellosis is previously endemic in these countries but is now primarily related to returning travelers [2]. Huge economic losses can still be caused by brucellosis in developing countries [3]. Although the mortality of brucellosis in humans is less than 1%, it can still cause severe debilitation and disability [4]. Brucellosis is listed as a class II infectious disease by the Chinese Disease Prevention and Control of Livestock and Poultry and as a class II reportable infectious disease by the Chinese Centers for Disease Control and Prevention (CDC) [5]. Currently, human brucellosis is still a main public health problem that endangers the life and health of people in China.

Surveillance and early warning are critical for the detection of infectious disease outbreaks. Therefore, establishing epidemiological models and conducting predictions are useful for the prevention and control of human brucellosis. Autoregressive Integrated Moving Average (ARIMA) models are time domain methods in time series analysis and have been widely used in infectious diseases forecasting [6–10]. ARIMA models can capture the long-term trends and the periodic variations in time series [6]. However, the models cannot handle the nonlinear trends correctly [11, 12]. In contrast, neural networks are flexible and nonlinear tools capable of approximating any kind of arbitrary function [13–15]. Recurrent neural networks (RNNs) can address problems that involve time series data. These models have been mainly used in adaptive control, system identification, and most famously in speech recognition. Elman and Jordan neural networks are two popular recurrent neural networks that have delivered outstanding performances in a wide range of applications [16–20]. Currently, no researchers have used these two neural networks to forecast the time series data of human brucellosis.

In this study, we build prediction models for human brucellosis in mainland China by using Elman and Jordan neural networks. In addition, the fitting and forecasting accuracy of the neural networks were compared with an Autoregressive Integrated Moving Average model.

## Methods

### Data sources

The reported human brucellosis cases were obtained from the website of the National Health and Family Planning Commission (NHFPC) of China (http://www.nhc.gov.cn). All of the human brucellosis cases were diagnosed according to clinical symptoms such as undulant fevers, sweating, nausea, vomiting, myalgia, arthralgia, an enlarged liver, and an enlarged spleen [21]. Additionally, the human brucellosis cases were also confirmed by a serologic test in terms of the case definition of the World Health Organization (WHO). The human brucellosis cases from January 2004 to December 2017 were assembled as monthly counts. The dataset analyzed during the study is included in Additional file 1. The dataset was split into two sections: a training set and a test set. The training set observed from January 2004 to December 2016 was used to build models, and the test set observed from January 2017 to December 2017 was used to test the forecast results.

### Decomposition of the time series

We first plotted the time series of human brucellosis cases and looked for trend and seasonal variations. One of the assumptions of an ARIMA model is that the time series should be stationary. The mean, variance, and autocorrelation of a stationary time series are constant over time. Logarithm and square root transformation of the original series were performed to stabilize the variance. A first-order difference and a seasonal difference were used to stabilize the long-term trend and seasonal variance, respectively. An augmented Dickey-Fuller (ADF) test was performed to check the stationary of the transformed time series.

We used an additive model to decompose the time series. An additive decomposition model was used as follows:

$$ {x}_t={m}_t+{s}_t+{z}_t $$

where at time t, x_t_ is the observed series, m_t_ is the trend, s_t_ is the seasonal effect, and z_t_ is an error term. The smooth algorithm used in this study is loess [22]. A locally weighted regression technique is used in this method.

### The seasonal ARIMA model construction

In the early 1970s, ARIMA models were proposed by statisticians Box and Jenkins and have been considered to be one of the most widely used models for time series analysis [23]. Autoregressive and moving average terms at lag s are included in a seasonal ARIMA model. The seasonal ARIMA(p, d, q) (P, D, Q)_s_ model is written with the backward shift operator as follows:

$$ {\Theta}_P\left({B}^s\right){\theta}_p(B){\left(1-{B}^s\right)}^D{\left(1-B\right)}^d{x}_t={\Phi}_Q\left({B}^s\right){\phi}_q(B){w}_t $$

where Θ_*P*_, *θ*_*p*_, Φ_*Q*_, and *ϕ*_*q*_ are polynomials of orders P, p, Q, and q, respectively. After a time series has been transformed to be stationary, the figures of the autocorrelation function (ACF) and partial autocorrelation function (PACF) are used to give a rough guide of reasonable models to try. Once the model order has been identified, a parameter test is necessary. We need to estimate the coefficients of autoregressive and moving average terms. The maximum likelihood estimation (MLE) is used to perform the parameter test. A best-fitting model is chosen with an appropriate criterion after trying out a wide range of models. A Ljung-Box Q statistic of the residuals is always used to judge whether the residuals are white noise. This statistic is a one-tailed statistical test. If the *p* value is greater than the significance level, then the time series is regarded as white noise. A Brock-Dechert-Scheinkman (BDS) test is applied to the residuals of the best-fitting seasonal ARIMA model to detect the nonlinearity of the original time series. A BDS test can test nonlinearity, provided that any linear dependence has been removed from the data. In this study, we wrote a function with R language that could fit a range of likely candidate ARIMA models automatically. The R script of the function is included in Additional file 2. The conditional sum of squares (CSS) method which was more robust was used in the arima function. The consistent Akaike Information Criteria (CAIC) [24] was used to select the best-fitting model. The formula of CAIC is written as follows:

$$ CAIC=-2 LL+\left[\mathit{\ln}(n)+1\right]K $$

where LL is the model log likelihood estimate, K is the number of model parameters, and n is the sample size. Good models are obtained by minimizing the CAIC.

### Building Elman and Jordan recurrent neural networks

Normalization is an important procedure in building a neural network, as it avoids unnecessary results or difficult training processes resulting in algorithm convergence problems. We used the min-max method to obtain all the scaled data between zero and one. The formula for the min-max method is the following:

$$ {x}_{scaled}=\frac{x-{x}_{min}}{x_{max}-{x}_{min}} $$

Since the time series data of human brucellosis cases had strong seasonality characteristics that appeared to depend on the month, we created twelve time-lagged variables as input features. Therefore, we selected twelve as the number of input neurons. There was one output neuron representing the forecast value of the cases of the next month. Supposing that x_t_ represents the human brucellosis cases at time t, then the input matrix and the output matrix of the modeling dataset used in this study are written as follows:

$$ input\ matrix=\left[\begin{array}{c}{x}_1\ {x}_2\cdots {x}_{12}\\ {}{x}_2\kern0.50em {x}_3\cdots {x}_{13}\\ {}\cdots \cdots \cdots {x}_{14}\\ {}\ {x}_{t-12}\ \ {x}_{t-11}\cdots {x}_{t-1}\end{array}\right] $$

$$ output\ matrix=\left[\begin{array}{c}{x}_{13}\\ {}{x}_{14}\\ {}\cdots \\ {}{x}_t\end{array}\right] $$

**Fig. 1:**
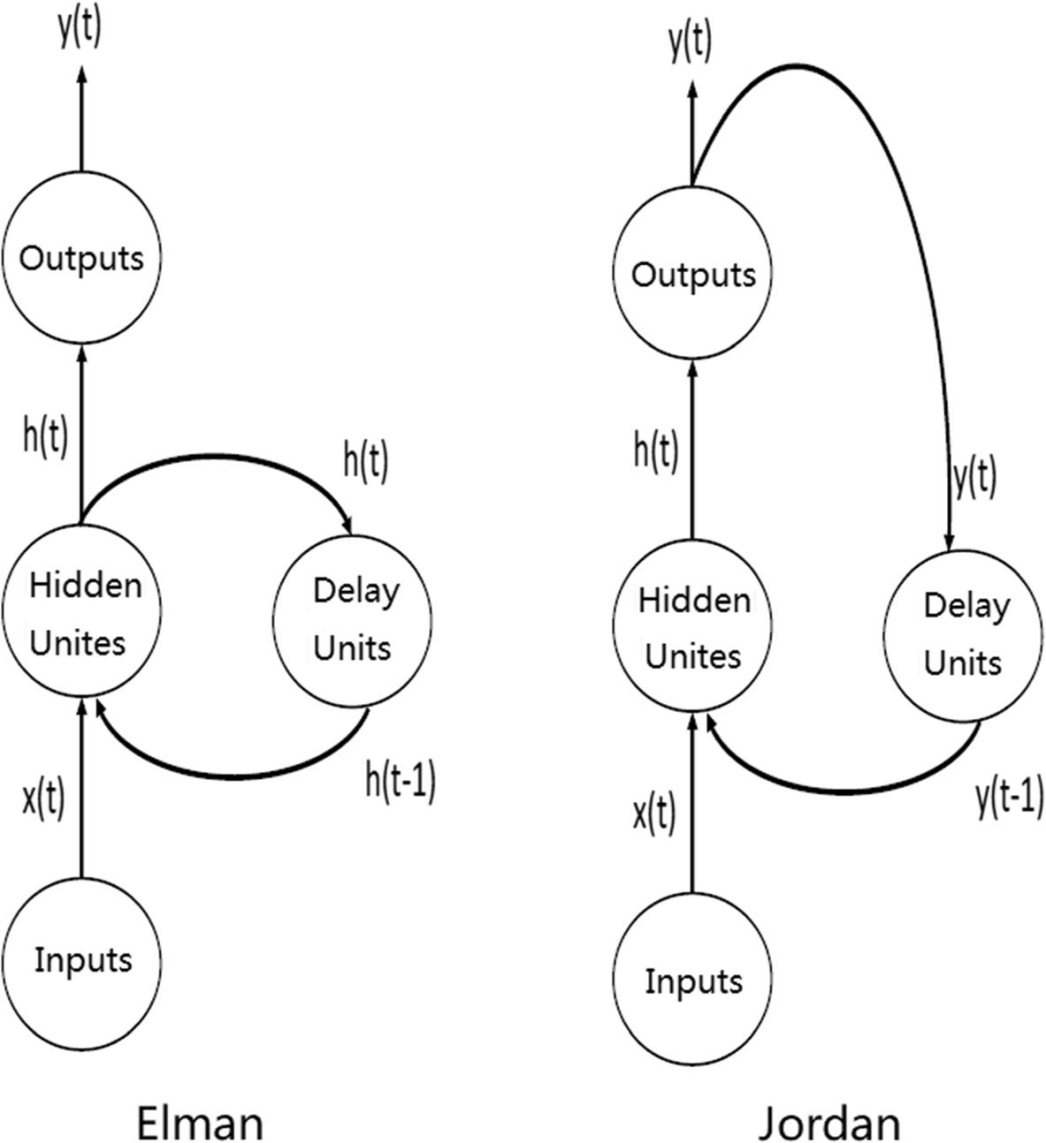
Structure of the Elman and Jordan neural networks

The structure of Elman and Jordan neural networks is illustrated in Fig. 1. Elman and Jordan neural networks consist of an input layer, a hidden layer, a delay layer, and an output layer. The delay neurons of an Elman neural network are fed from the hidden layer, while the delay neurons of a Jordan neural network are fed from the output layer.

There are several parameters, such as the number of units in the hidden layer, the maximum number of iterations to learn, the initialization function, the learning function, and the update function, which should be set when we build an Elman or Jordan neural network. In this study, the maximum number of iterations was set to 5000. If the learning rate is too large, then the neural network will converge to local minima. Therefore, the learning rate is usually between 0 and 1. By trial and error, the learning rates of Elman and Jordan neural networks were set to 0.75 and 0.55, respectively. One hidden layer was sufficient for this study. To avoid over fitting during the training process, we adopted the approach of leave-one-out-cross-validation (LOOCV) in the original training set for selecting the optimal number of units in the hidden layer. A single observation was used for the validation set, and the remaining observations made up the new training set. Then, we trained the neural network on the remaining ones and computed the mean squared error (MSE) for the selected single network. We repeated the procedure until every observation had been selected once in the original training set. The number of units in the hidden layer was attempted from 5 to 25. The optimal number of units in the hidden layer had the lowest mean MSE. The parallel computation was used to accelerate the LOOCV procedure on a Dell PowerEdge server T430 with 12 threads. The remaining parameters of the two models were set to default. The R script to conduct the neural network models is included in Additional file 3.

### Model comparison

Three performance indexes, root mean squared error (RMSE), mean absolute error (MAE) and mean absolute percentage error (MAPE), were used to assess the fitting and forecasting accuracy of the three models. MAE is the simplest measure of fitting and forecasting accuracy. We can calculate the absolute error with the absolute value of the difference between the actual value and the predicted value. MAE determines how large of an error we can expect from the forecast on average. To address the problem of telling a large error from a small error, we can find the mean absolute error in percentage terms. MAPE is calculated as the average of the unsigned percentage error. The MAPE is scale sensitive and should not be used when working with low-volume data. Since MAE and MAPE are based on the mean error, they may understate the impact of large rare errors. RMSE is calculated to adjust for large rare errors. We first square the errors, then calculate the mean of errors and take the square root of the mean. We can obtain a measure of the size of the error that gives more weight to the large rare errors. We can also compare RMSE and MAE to judge whether the forecast contains large rare errors. Generally, the larger the difference between RMSE and MAE, the more inconsistent the error size is.

### Data analysis

All data analyses were conducted by using R software version 3.5.1 on an Ubuntu 18.04 operating system. The decomposition of the time series was performed with the function stl in package stats. Seasonal ARIMA models were built with the function arima in package stats. Elman and Jordan recurrent neural networks were built with the functions elman and jordan in the RSNNS package, respectively. In this study, the statistical significance level was set at 0.05.

## Results

### Characteristics of human brucellosis cases in mainland China

**Fig. 2:**
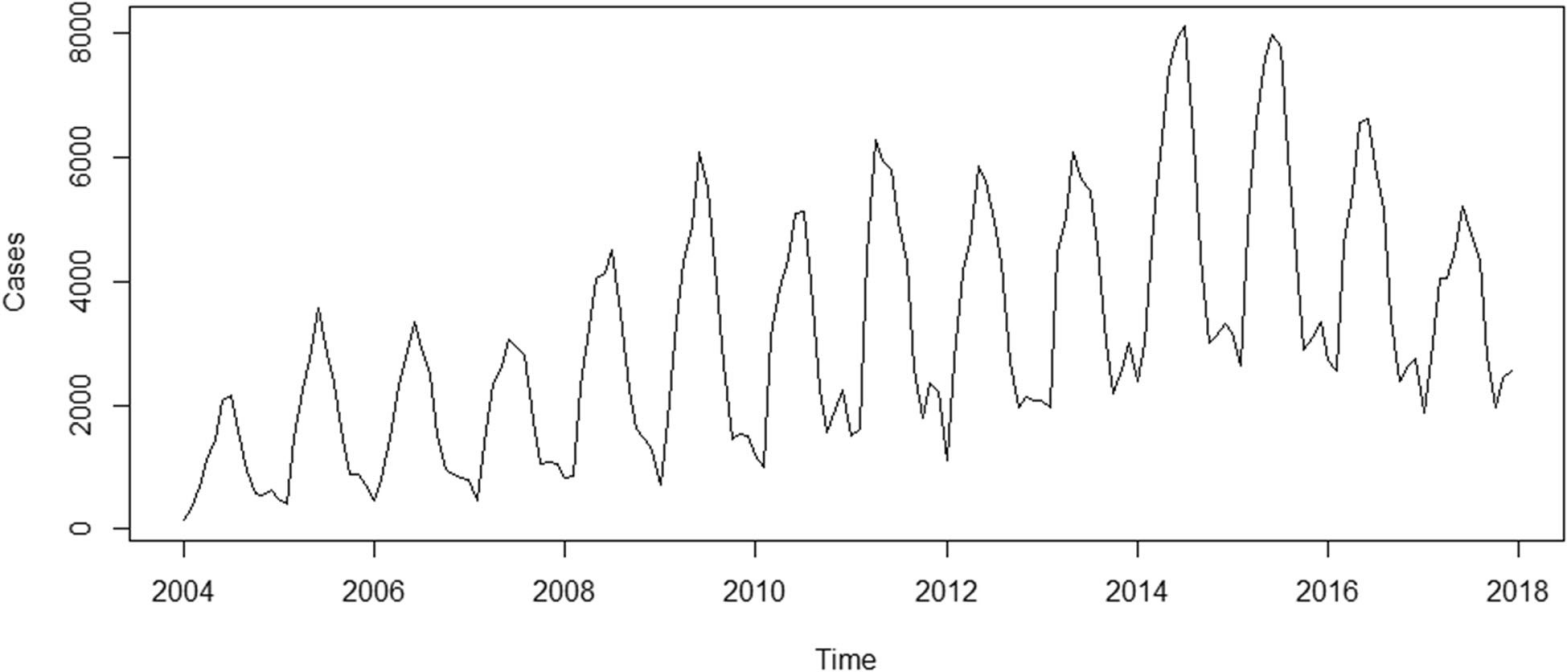
Time series plot for cases of human brucellosis in Mainland China from 2004 to 2017

**Fig. 3:**
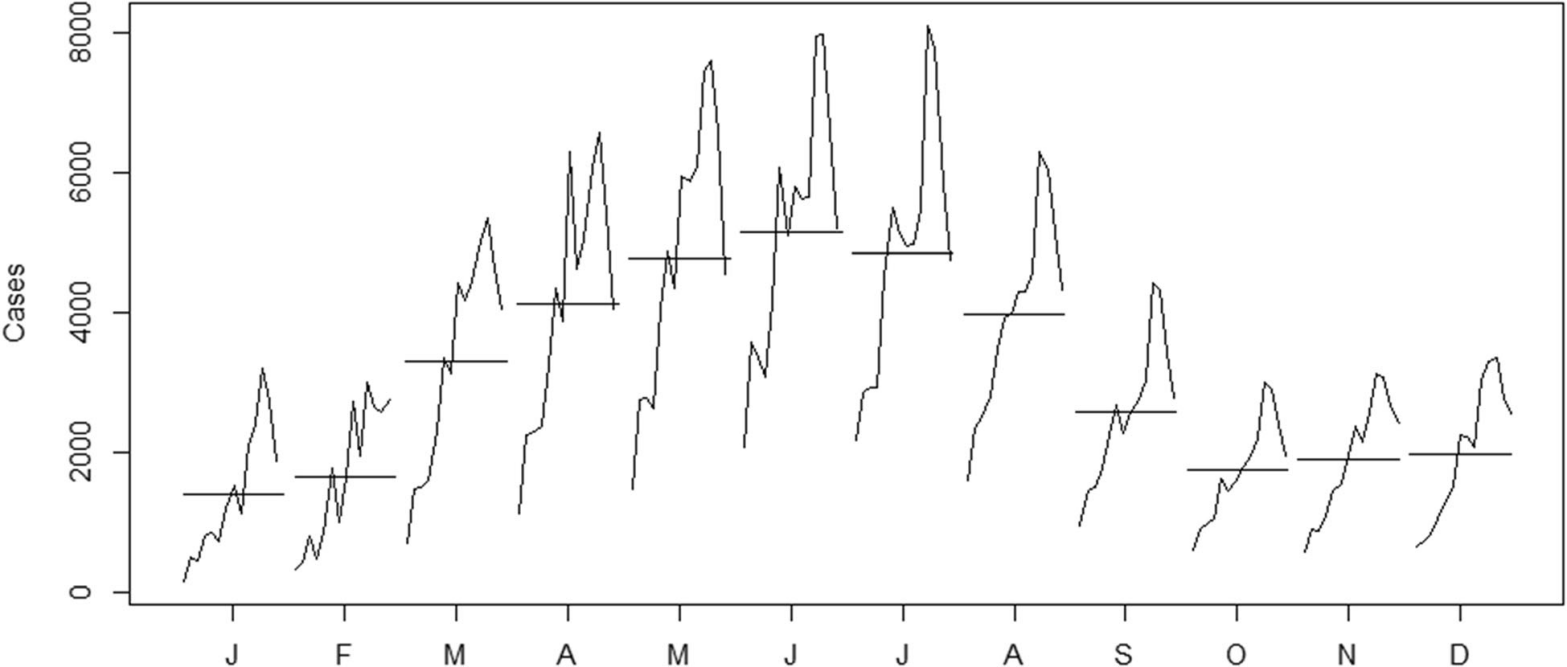
Month plot for cases of human brucellosis in Mainland China from 2004 to 2017

**Fig. 4:**
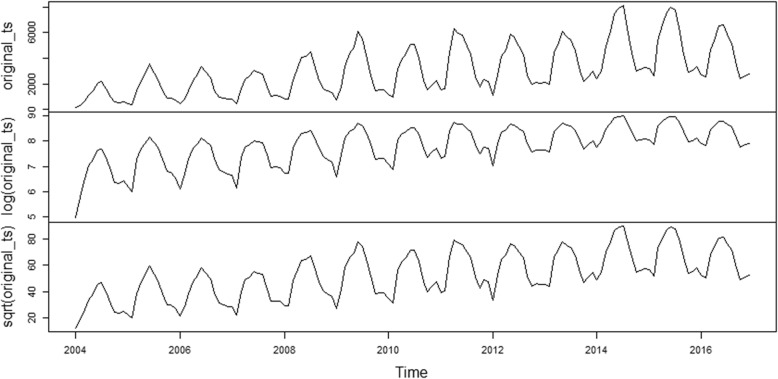
Plot of original time series, logarithm and square root transformed human brucellosis cases

**Fig. 5:**
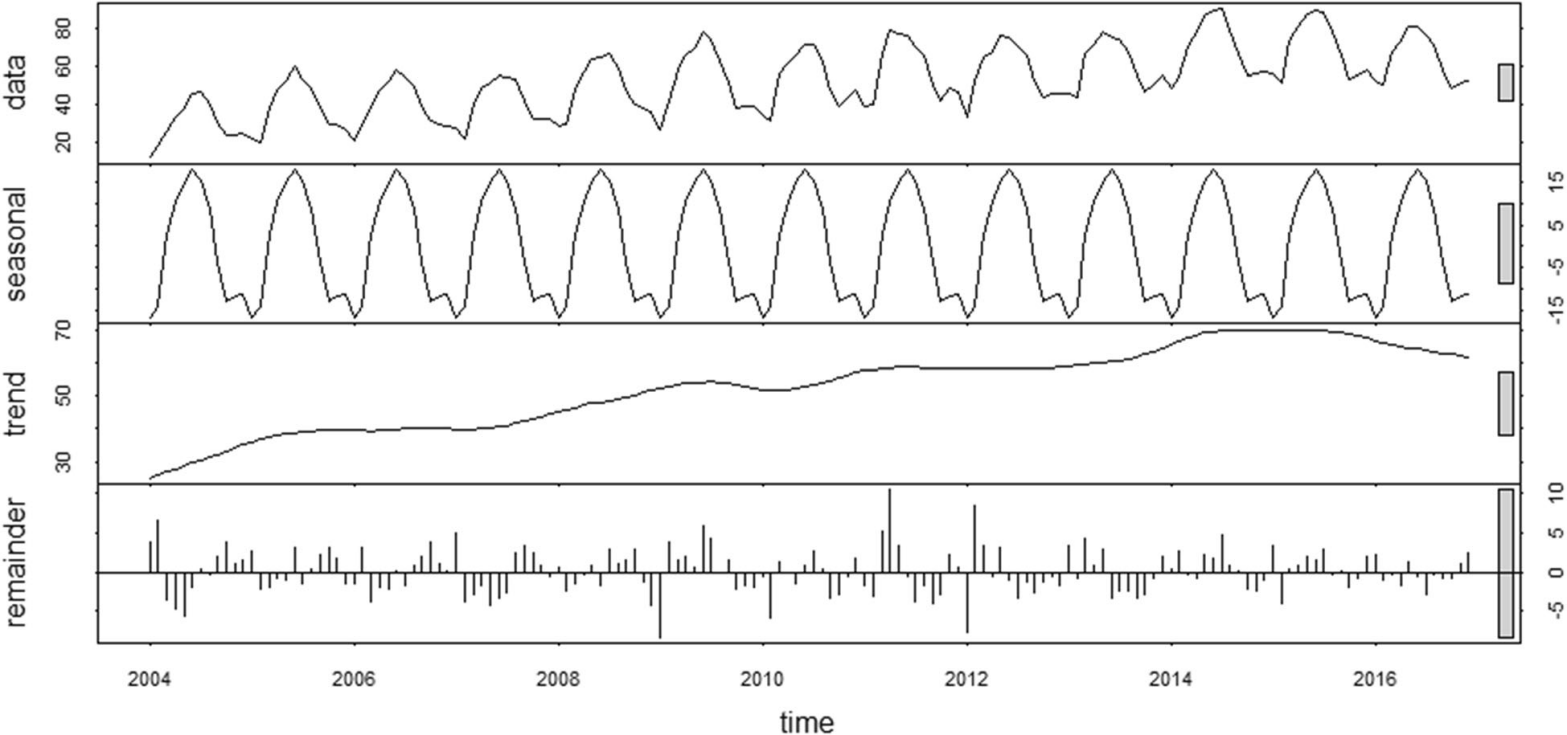
Seasonal decomposition of the square root transformed human brucellosis cases

There were 52,868 cases of human brucellosis in Mainland China from January 2004 to December 2017. As shown in Fig. 2, we can observe a general upward trend and seasonal variance in the original time series. The number of human brucellosis cases was higher in summer and lower in winter (Fig. 3). As shown in Fig. 2, the variance seemed to increase with the level of the time series. The variance from 2004 to 2007 was the smallest, followed by the data from 2008 to 2013, and the variance from 2014 to 2016 was the largest. Therefore, the variance was nonconstant. The time series must be transformed to stabilize the variance. Generally, the logarithm or square root transformation can stabilize the variance. The plot of the original time series, logarithm and square root transformed human brucellosis case time series is illustrated in Fig. 4. The variance seemed to decrease with the level of the logarithm transformed human brucellosis case time series. For the square root transformed human brucellosis cases time series, the variance appeared to be fairly consistent. We found that square root transformation was more appropriate for this study after trying these two methods. The decomposition of time series after square root transformation is plotted in Fig. 5. The gray bars on the right side of the plot allowed for the easy comparison of the magnitudes of each component. The square root transformed time series, seasonal, trend, and noise components are shown from top to bottom, respectively. The seasonal component did not change over time. The trend component showed a general upward trend from 2004 to 2015 and declined slightly in 2016. There was no apparent pattern of noise. The results of the decomposition were satisfactory.

### Seasonal ARIMA model

**Fig. 6:**
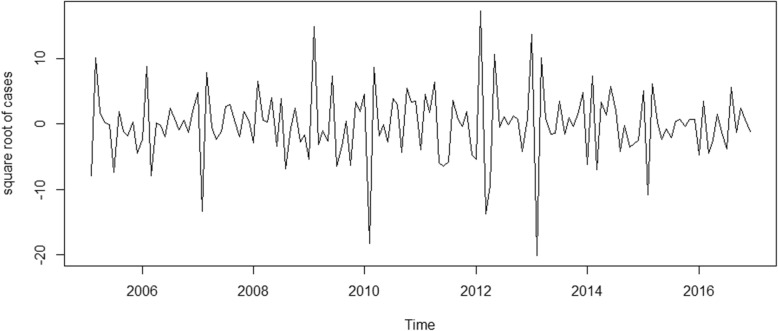
Plot of square root transformed human brucellosis cases after a first-order difference and a seasonal difference

A first-order difference and a seasonal difference made the time series of square root transformed human brucellosis cases look relatively stationary (Fig. 6). The ADF test of the differenced time series suggested that it was stationary (ADF test: t = − 5.327, *P* < 0.01). Therefore, the parameters d and D for a seasonal ARIMA model were set to 1 and 1, respectively.

**Fig. 7:**
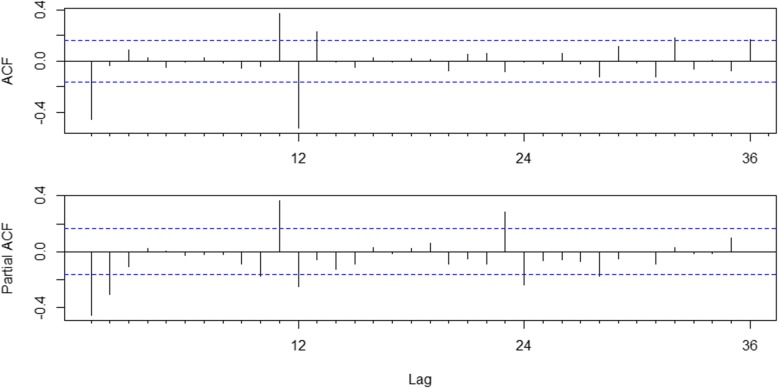
Autocorrelation and partial autocorrelation plots for the differenced stationary time series

The plots of ACF and PACF are shown in Fig. 7. The significant spike at lag 1 in the ACF suggested a possible nonseasonal MA (1) component, and the significant spike at lag 12 in the ACF suggested a possible seasonal MA (1) component. The significant spikes at lags 1 and 2 in the PACF suggested a possible nonseasonal AR (2) component, and the significant spikes at lags 12 and 24 in the PACF suggested a possible seasonal AR (2) component. Therefore, we tried the parameters p from 0 to 2, q from 0 to 1, P from 0 to 2, and Q from 0 to 1. With the combination of these parameters, 36 likely candidate models were built. The models were considered as to whether they could pass residual and parameter tests. Eventually, five models remained. We found that the ARIMA (2,1,0) × (0,1,1)_12_ model had the smallest CAIC (798.731) among the candidate models (Table 1). The Ljung-Box Q statistic of the residuals indicated no significant difference (*P* = 0.097) at the significance level of 0.05. Therefore, we considered that the residuals were white noise. The estimated parameters of the optimal seasonal ARIMA model are listed in Table 2. The results of the BDS test are shown in Table 3, and all of the *p* values were smaller than the significance level of 0.05. The results suggested that the time series of human brucellosis in Mainland China from 2004 to 2016 was not linear.

**Table 1:** Comparison of five candidate seasonal ARIMA models

Model	CAIC	Ljung-Box Q	P value
ARIMA (0,1,1) × (0,1,1)_12_	799.923	22.787	0.199
ARIMA (0,1,1) × (1,1,0)_12_	817.906	22.472	0.212
ARIMA (0,1,1) × (2,1,0)_12_	813.588	25.848	0.103
ARIMA (2,1,0) × (0,1,1)_12_	798.731	26.107	0.097
ARIMA (2,1,0) × (1,1,0)_12_	823.300	22.852	0.196

**Table 2:** Estimate parameters of the seasonal ARIMA (2,1,0) × (0,1,1)_12_ model

Model parameter	Estimate	Standard error	95%CI of estimate
AR1	−0.392	0.085	(−0.559, − 0.225)
AR2	−0.220	0.081	(−0.378, − 0.062)
Seasonal MA1	− 0.726	0.063	(− 0.849, − 0.603)

**Table 3:** Results of BDS test for the residuals of seasonal ARIMA model

Epsilon	Dimension	Statistic	*p*-value
1.838	2	2.751	0.006
1.838	3	3.532	0.000
1.838	4	2.997	0.002
3.677	2	2.241	0.025
3.677	3	2.603	0.009
3.677	4	2.524	0.012
5.515	2	2.371	0.018
5.515	3	2.452	0.014
5.515	4	2.317	0.021
7.354	2	2.624	0.009
7.354	3	2.572	0.010
7.354	4	2.472	0.013

### Elman and Jordan neural networks

**Fig. 8:**
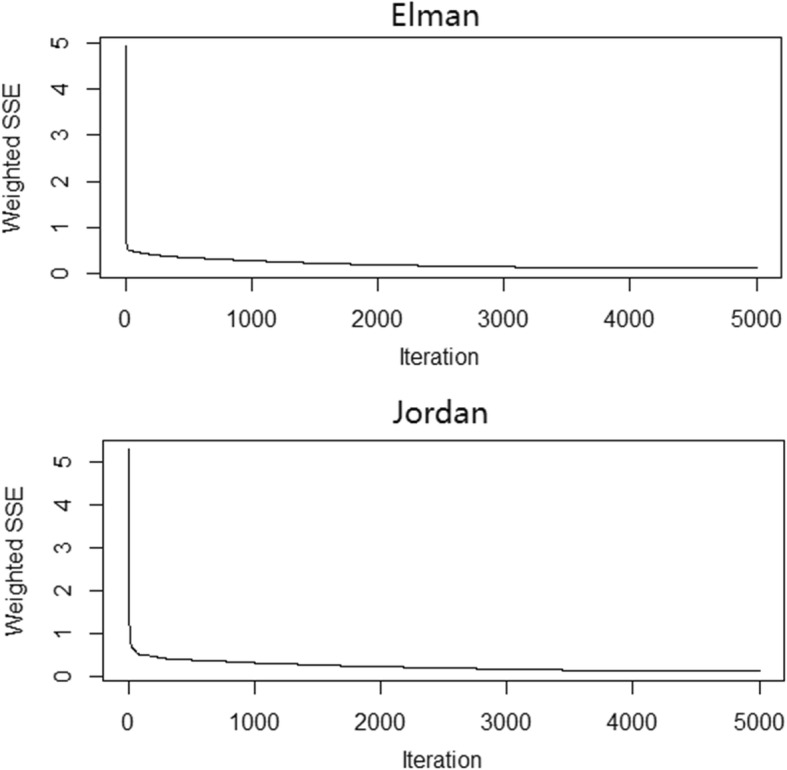
Training error by iteration for Elman and Jordan neural networks

When we used the LOOCV approach in the training set, the lowest mean MSE was 0.018 and 0.010 for Elman and Jordan neural networks when the number of units in the hidden layer was 7 and 8, respectively. The plot of the training error by iteration is shown in Fig. 8. The error dropped sharply within the beginning iterations. This finding indicated that the model was learning from the data. The error then declined at a more modest rate until 5000 iterations.

### Comparison of the three models

The training set was used to build models. A first-order difference and a seasonal difference were performed in building the seasonal ARIMA model. Therefore, we lost the first 13 values in the training set, and the remaining 143 values were compared. We created 12 time-lagged variables as input features for Elman and Jordan neural networks. Therefore, 144 values were compared in the training set for the neural networks. The fitting and forecasting accuracy of the three models are shown in Table 4. In the training set, the RMSE and MAE of Elman and Jordan neural networks were lower than those of the ARIMA model, whereas the MAPE of Elman and Jordan neural networks was slightly higher than that of the ARIMA model. In the test set, the RMSE, MAE and MAPE of the Elman and Jordan neural networks were far lower than those of the ARIMA model. The Jordan neural network had the best forecasting performance. The RMSE, MAE and MAPE of the Jordan neural network were the lowest. Therefore, the Jordan neural network was the best model for the test set. The actual and forecasted cases of human brucellosis in mainland China from January to December 2017 of the three models are presented in Table 5.

**Table 4:** Comparison of the fitting and forecasting accuracy of the three models

performance index	Training set				Test set		
	ARIMA	Elman	Jordan		ARIMA	Elman	Jordan
RMSE	405.746	297.181	361.283		1050.018	684.450	561.442
MAE	294.190	231.061	287.370		873.840	502.926	374.737
MAPE	0.112	0.115	0.113		0.236	0.156	0.113

**Table 5:** The actual and forecasted cases of human brucellosis in mainland China from January to December 2017 of the three models

Month	Actual values	ARIMA	Elman	Jordan
Jan	1874	2274	2148	2140
Feb	2740	2390	2395	2399
Mar	4055	4568	4211	4267
Apr	4048	5530	5077	5376
May	4539	6521	6085	5763
Jun	5203	6721	4450	5175
Jul	4742	6330	4873	4795
Aug	4330	5228	4376	4421
Sep	2781	3527	3478	3141
Oct	1953	2448	2890	2269
Nov	2427	2675	2489	2361
Dec	2549	2819	2612	2337

## Discussion

There are two methods for time series analysis: frequency domain methods and time domain methods. Seasonal ARIMA models, which belong to time domain methods, have been regarded as one of the most useful models in seasonal time series prediction [25]. There is no need to use any extra surrogate variables [26]. We can usually only analyze with the outcome variable series without considering the factors that will affect the outcome variable. This method is more practical because we cannot obtain all of the time series data of impacting factors most of the time. Before the model identification, the time series should be handled to be stationary with data transformation and difference. Generally, the more differences are used, the more data loss will occur. Fortunately, we only used a first-order difference and a seasonal difference in this study. Eventually, the ARIMA (2,1,0) × (0,1,1)_12_ model was chosen as the optimal model according to the value of CAIC. The seasonal ARIMA model accurately captured the seasonal fluctuation of human brucellosis cases in mainland China. However, the forecasting accuracy in the test set was not satisfactory. The MAPE of the seasonal ARIMA model reached 0.236 in the test set. The most likely reason was that the time series data of human brucellosis cases in mainland China was not linear. As shown in Fig. 5, although we could observe a long-term upward trend from the trend component, some curves remained after the seasonal and irregular components had been extracted. The results of the BDS test also supported that the time series of human brucellosis in Mainland China from 2004 to 2016 was not linear.

There are mainly two approaches for nonlinear time series forecasting [27]. One approach is model-based parametric nonlinear methods, such as the smoothing transition autoregressive (STAR) model, the threshold autoregressive (TAR) model, the nonlinear autoregressive (NAR) model, the nonlinear moving average (NMA) model, etc. In theory, these parametric nonlinear methods are superior to the traditional ARIMA model in capturing nonlinear relationships in the data. However, there are too many possible nonlinear patterns in practice, which restricts the usefulness of these models. The other approach is nonparametric data driven methods, and the most widely used method is neural networks. Neural networks are inspired by the structure of a biological nervous system. These networks can capture the patterns and hidden functional relationships existing in a given set of data, although these relationships are unknown or hard to identify [28]. Recurrent neural networks contain hidden states that are distributed across time. This characteristic suggests that these networks have the ability to efficiently store much information about the past. Therefore, these networks have the advantage of dealing with time series data. Elman and Jordan neural networks are two widely used recurrent neural networks. Elman neural networks have been used in many practical applications, such as the price prediction of crude oil futures [29], weather forecasting [30], water quality forecasting [31], and financial time series prediction [28]. Jordan neural networks have been used in wind speed forecasting [32] and stock market volatility monitoring [33]. All of these applications have achieved good forecasting performance. In this study, we tried these two neural network models to predict human brucellosis cases in Mainland China. The MAPE of Elman and Jordan neural networks were 0.115 and 0.113, respectively, almost the same as the MAPE of the seasonal ARIMA model at 0.112 in the training set, while the RMSE and MAE of Elman and Jordan neural networks were lower than those of the ARIMA model. The RMSE and MAE of the Elman neural network were the lowest, whereas the MAPE of the Elman neural network was the highest in the training set. The most likely reason was that the Elman neural network gained better fitting accuracy for large values, but gained poorer fitting accuracy for small values in this study. Importantly, the Elman and Jordan neural networks achieved much higher forecasting accuracy in the test set. The RMSE, MAE, and MAPE of Elman and Jordan neural networks were far lower than those of the seasonal ARIMA model. Therefore, Elman and Jordan Recurrent Neural Networks are more appropriate than the seasonal ARIMA model for forecasting nonlinear time series data, such as human brucellosis. However, we must admit that there are still some limitations of neural network models. First, neural network models are black boxes, i.e., we cannot know how much each input variable is influencing the output variables. Second, there are no fix rules to determine the structure and parameters of neural network models. It all depends on the experience of researchers. Third, it is computationally very expensive and time consuming to train neural network models. Neural network models require processors with parallel processing power to accelerate the training process. Some researchers have built hybrid models combining ARIMA models and neural network models to analyze time series data and achieved good results. We will try hybrid models for human brucellosis in the future. There were still some limitations to this study. First, the NHFPC of China only reported the data from 2004 to 2017. More time series data on brucellosis cases can improve the accuracy of forecasting models. Second, the present study is an ecological study, and we cannot avoid ecological fallacy. Third, the factors that affect the occurrence of human brucellosis such as pathogens, host, natural environment, vaccines and socioeconomic variations were not considered when we conducted the models.

## Conclusions

In this study, we established a seasonal ARIMA model and two recurrent neural networks, namely, the Elman and Jordan neural networks, to conduct short-term prediction of human brucellosis cases in mainland China. The Elman and Jordan recurrent neural networks achieved much higher forecasting accuracy. These models are more appropriate for forecasting nonlinear time series data, such as human brucellosis, than the traditional ARIMA model.

## Additional files

Additional file 1: Monthly cases of human brucellosis in Mainland China from 2004 to 2017. (XLSX 13 kb)

Additional file 2: R script of the function that can fit a range of likely candidate ARIMA models automatically. (TXT 1 kb)

Additional file 3: R script to perform the Elman and Jordan neural network models with leave-one-out-cross-validation method. (TXT 2 kb)

## Abbreviations

ACF: Autocorrelation function
ADF: Augmented Dickey-Fuller
ARIMA: Autoregressive Integrated Moving Average
BDS: Brock-Dechert-Scheinkman
CAIC: Consistent Akaike Information Criteria
CDC: Centers for Disease Control and Prevention
CSS: Conditional sum of squares
LOOCV: Leave-one-out-cross-validation
MAE: Mean absolute error
MAPE: Mean absolute percentage error
MLE: Maximum likelihood estimation
MSE: Mean squared error
NAR: Nonlinear autoregressive
NHFPC: National Health and Family Planning Commission
NMA: Nonlinear moving average
PACF: Partial autocorrelation function
RMSE: Root mean squared error
RNNs: Recurrent Neural Networks
STAR: Smoothing transition autoregressive
TAR: Threshold autoregressive
WHO: World Health Organization
